# Maternal supplementation with *Limosilactobacillus reuteri* FN041 for preventing infants with atopic dermatitis: study protocol for a randomized controlled trial

**DOI:** 10.3389/fmicb.2023.1267448

**Published:** 2023-10-05

**Authors:** Renqiang Yu, Yizhe Ma, Zichen Luo, Ce Qi, Anni Xie, Yifan Jiang, Baoli Zhu, Jin Sun

**Affiliations:** ^1^Department of Neonatology, Wuxi Maternity and Child Health Care Hospital, Women’s Hospital of Jiangnan University, Wuxi, China; ^2^Department of Pediatric, Jiangyin People's Hospital of Nantong University, Wuxi, China; ^3^Institute of Nutrition and Health, Qingdao University, Qingdao, China; ^4^School of Medicine, Nantong University, Nantong, China

**Keywords:** atopic dermatitis, *Limosilactobacillus reuteri*, infant, microbiota, breast milk, probiotics

## Abstract

**Background:**

Atopic dermatitis (AD) has increased rapidly with rapid urbanization; however, the treatment options for AD are lacking because the commonly used therapies can only alleviate symptoms. *Limosilactobacillus reuteri (L. reuteri),* FN041 is a specific strain isolated from human breast milk, and its protective potential against AD has been confirmed. This study aims to assess the efficacy of maternal consumption of *L. reuteri* FN041 during late pregnancy and lactation in preventing infantile AD.

**Methods:**

First, a randomized, double-blind, placebo-controlled intervention study will be conducted on 340 pregnant females with babies at high risk for AD. These subjects will be randomly divided into four groups of different doses of *L. reuteri* FN041 (1 × 10^9^, 5 × 10^9^, and 1 × 10^10^ CFU/d) along with a placebo. The safety and efficacy of maternal use of *L. reuteri* FN041 for preventing infantile AD will be analyzed, and the most efficient dosage of *L. reuteri* FN041 will be determined. Subsequently, a multicenter cohort study of 500 pregnant females with babies at high risk for AD will be conducted to promote the maternal application of *L. reuteri* FN041. These subjects will be administered *L. reuteri* FN041 at the optimal dose determined during the first stage of late pregnancy and lactation, and their babies will be analyzed for AD development. Recruitment was initiated in October 2022.

**Discussion:**

The primary outcome is the cumulative incidence of AD at 24 months after maternal consumption of *L. reuteri* FN041 during late pregnancy and lactation, whereas the secondary outcome is the efficiency of *L. reuteri* FN041 transfer from the mother’s gut to breast milk and then the infant’s gut after oral supplementation. This study will demonstrate the efficacy of edible probiotics isolated from breast milk in preventing or treating AD in infants. Accordingly, we provide population-based advice for administering specific probiotics for the primary prevention of AD in pregnant females. Understanding the underlying mechanisms of probiotic strains derived from breast milk can promote their application in preventing infant diseases associated with intestinal microbiota imbalance and immune disorders.

**Clinical trial registration:**

https://www.chictr.org.cn/, identifier [ChiCTR2300075611].

## Introduction

### Background and rationale

Atopic dermatitis (AD) is an immune-associated inflammatory skin disease characterized by recurrent severe itching and dry skin (Langan et al., 2020). Severe itching affects sleep, growth, and development of young children (Nicholas et al., 2022; Wollenberg et al., 2023). Without effective intervention, infants with AD are susceptible to developing asthma, allergic rhinitis, and conjunctivitis (Elezbawy et al., 2023). The prevalence of AD among urban infants in China is rapidly increasing and similar conditions have been observed in other developing countries (Elezbawy et al., 2023). AD usually occurs early in life. In China in 2019, the total prevalence of AD in infants aged 1–12 months was 30.48%; among which, the incidence in infants aged 3 months was approximately 40.81% (Guo et al., 2019). Currently, clinical therapy commonly involves the use of glucocorticoids and immunosuppressive drugs; however, these drugs alleviate the clinical symptoms of AD only to a certain extent and may cause side effects (Wollenberg et al., 2023). Further, treatment with drugs is challenging in terms of limitations in drug selection for infants and young children (Wollenberg et al., 2023). Therefore, it is necessary to find suitable prevention and therapeutic strategies for infants and young children with AD.

Infant gut microbiota colonization and abnormal immune response are involved in the development of AD (Mahmud et al., 2022). In children with AD, the diversity of the gut microbiota and relative abundance of *Lactobacilli* and *Bifidobacteria* are substantially decreased, whereas the abundance of pathogenic bacteria such as *Escherichia coli*, *Clostridium difficile*, and *Staphylococcus aureus* is notably increased, compared to healthy individuals (Kim and Kim, 2019). Alterations in the gut bacterial community are correlated with disease severity (Kim and Kim, 2019). Previous data have revealed that exclusive breastfeeding in the first 3 months of life is associated with a lower incidence of AD in infants with a family history of allergic diseases (Gdalevich et al., 2001). This protective effect may be related to the microorganisms supplied by breast milk, which ensures a continuous supply of bacteria throughout the lactation period for infants (Lyons et al., 2020).

Interestingly, a recent meta-analysis summarized 21 relevant randomized controlled trials conducted in different countries and confirmed that supplementing mothers with composite probiotics before and after delivery can prevent infantile AD, whereas supplementing infants with probiotics may not effectively prevent the onset of AD (Amalia et al., 2020). Among these studies, five observed that probiotic mixture interventions during pregnancy and lactation reduced the incidence of AD in infants by approximately 28% (Amalia et al., 2020). However, in studies using a single probiotic strain, *lactobacillus rhamnosus* HN001 (HN001) or *lactobacillus rhamnosus* GG (LGG), for maternal supplementation did not reduce the incidence rate of AD in infants (Ou et al., 2012; Wickens et al., 2018). However, supplementation of both mothers and infants with HN001 during pregnancy and lactation can significantly reduce the incidence of AD by 2 years (Wickens et al., 2008). The main reason behind the poor preventive effects of probiotics against AD when administered to mothers during pregnancy and lactation, rather than to infants, may be explained by the fact that probiotics such as LGG cannot be effectively transmitted through breast milk to the infant’s intestines (Simpson et al., 2018). The key to preventing AD in infants via maternal consumption of probiotics is to use strains that can be transmitted from the mother’s gut to breast milk.

The infant gut microbiota changes rapidly in early life and gradually shifts to the adult composition during the first year after birth (Pickard et al., 2017). Breastfeeding has been suggested to be a key factor in establishing the infant gut microbiome (Baldassarre et al., 2018). According to entero-mammary pathway hypothesis, some bacteria in the mother’s gut can be transported to the breast through the lymphatic system and enter the infant’s gut via breast milk (Rodriguez, 2014). Our previous work demonstrated that secretory immunoglobulin A (sIgA)-coated bacteria are the key bacteria for vertical transmission from mother to child (Ding M. et al., 2022; Qi et al., 2022). In the agricultural and pastoral areas of the Gannan Tibetan Autonomous Prefecture of China, the relative abundance of *Limosilactobacillus reuteri* in human milk is far higher than that in other urbanized areas, which may contribute to the low incidence of allergic diseases (Chen, 2013; Ding et al., 2019). We previously used targeted sIgA immunomagnetic bead enrichment and separation technology to isolate a strain of sIgA-coated *L. reuteri* (isolation number FN041, patent strain storage number GDMCC60546) from the breast milk of Gannan females. This strain is included in the list of bacterium strains that can be used for food released by the National Health Commission and is a probiotic that can be used in ordinary food.

We also observed that maternal supplementation with *L. reuteri* FN041 during late gestation and lactation, along with offspring supplementation with *L. reuteri* FN041 after weaning, could effectively protect against AD (Zhao et al., 2022). In mice, the protective effect of maternal intervention with the sIgA-coated *L. reuteri* strain, which can be transmitted to milk, may modulate the intestinal microbiota community and the immune response of the offspring (Zhao et al., 2022; Qi et al., 2023). Moreover, further research has proven that the protective effect imparted by maternal supplementation with *L. reuteri* FN041 was much stronger than that imparted by the *L. reuteri* FN041 supplementation only in infant mice to prevent AD after weaning (Zhou et al., 2022). Therefore, assuming that maternal consumption of *L. reuteri* FN041 during pregnancy and lactation may be effective in preventing AD in offspring, we conducted a randomized controlled trial to provide data on this primary prevention strategy for AD.

### Objectives

This study aims to assess the incidence of infantile AD after maternal consumption of *L. reuteri* FN041 during late pregnancy and lactation. The optimum dosage will be determined by analyzing the efficacy of maternal supplementation with different doses of *L. reuteri* FN041 in preventing AD in offspring. Additionally, the secondary objective is to identify the transfer efficiency of *L. reuteri* FN041 from the mother’s gut to breast milk and then to infant’s gut after oral supplementation.

### Trial design

The study is divided into two stages. In the first stage, a multicenter, randomized, double-blind, placebo-controlled intervention study will be conducted in 340 pregnant females with babies at high risk for AD recruited from the Wuxi Maternity and Child Health Care Hospital affiliated with Jiangnan University and the Affiliated Hospital of Qingdao University. These subjects will be randomly divided into four groups (*n* = 85 for each group): (1) placebo group administered with the carrier solid beverage daily, (2) group administered with *L. reuteri* FN041 solid beverage at a dose of 1 × 10^9^ CFU/d, (3) group administered with *L. reuteri* FN041 solid beverage at a dose of 5 × 10^9^ CFU/d, and (4) group administered with *L. reuteri* FN041 solid beverage at a dose of 1 × 10^10^ CFU/d. Their offspring will be examined for AD development during the 2 years after birth so that the efficacy of maternal use of *L. reuteri* FN041 in the prevention of AD can be analyzed by calculating the cumulative incidence of AD in infants. Adverse reactions will be also recorded. Studies have reported that the most efficient dosage of *L. reuteri* FN041 can be determined within 6 months after birth, as infantile AD often develops in early life and peaks in the third month of life (Guo et al., 2019).

In the second stage of *L. reuteri* FN041 promotion and application, a multicenter cohort study in an expanded research population will be conducted. A total of 500 pregnant females with babies at a high risk for AD will be recruited from four tertiary hospitals: Wuxi Maternity and Child Health Care Hospital affiliated with Jiangnan University, Affiliated Hospital of Qingdao University, Suzhou Municipal Hospital, and Changzhou Maternal and Child Health Hospital. These subjects will be administered with *L. reuteri* FN041 at the optimal dosage determined in the first stage during late pregnancy and lactation; subsequently, their babies will be followed up for AD development for 2 years after birth.

## Methods

### Eligibility criteria

According to the World Allergy Organization guidelines, a child may be considered at high risk for allergy if a biological parent or sibling has a history of allergic rhinitis, asthma, eczema, or food allergy (Fiocchi et al., 2015). The recruited pregnant females with babies at high risk for AD must meet the following inclusion criteria: healthy pregnant female (14–16 weeks of gestation), the mother or father of the fetus, or previous child of the couple diagnosed with any of the allergic diseases, including asthma, eczema, food allergy, or allergic rhinitis. This information will be confirmed by specific recruiters prior to recruitment. The risk of developing AD in infants without a family history of allergies is approximately 27%, whereas that in infants with one or two parents with an atopic history increases to 37.9 and 50.0%, respectively (Böhme et al., 2003). The population we aim to recruit meets the recommended probiotic target audience according to the guidelines of the World Allergy Organization (Fiocchi et al., 2015). The exclusion criteria are as follows: pregnant female under 16 years of age, multiple pregnancies, known fetal abnormalities, oral administration of immunosuppressive drugs or antibiotics, heart valve disease or immunodeficiency, undergoing dental surgery under antibiotic prevention, history of transplantation or HIV, long-term continuous use or ongoing use or intent to use probiotics before admission, external fertilization pregnancy, previous participation in research, deemed unsuitable for inclusion in the study owing to other medical reasons, and gestational diabetes.

### Sample size calculation

The cumulative incidence of AD in high-risk infants at 1 and 2 years of age is reported to be approximately 40.1 and 41%, respectively, based on previous studies conducted in other countries (Rautava et al., 2012; Chaoimh et al., 2023). According to a previous study carried out in 2022 in our hospital, the overall point prevalence of AD in infants aged 0–24 months was 50.5% in Wuxi (Ding Y. et al., 2022). The expected cumulative incidence of AD after *L. reuteri* administration is approximately 20%, with a relative 20% reduction in AD incidence of 40% through intervention. Assuming that the first type of error would be 2.5% and the efficacy would be 80%, we calculated the sample using online tools.^1^ The sample size in each group would be 74. Considering a 10% dropout rate, each group should recruit 85 research subjects in the first stage. In the second stage of the trial, the sample size is expected to be approximately 500, which is considered sufficient to further verify the efficacy of maternal use of *L. reuteri* FN041 in the prevention of infantile AD.

### Informed consent

The researchers will screen and record the medical history of participants and obtain written informed consent from the recruited participants with a detailed explanation. On the informed consent form, the recruited subjects will be asked whether they agree to share their data and donate their breast milk, cord blood, and feces.

### Interventions

#### Intervention strategy

The *L. reuteri* strain used in this study is a probiotic supplement derived from breast milk and formulated in the form of a solid beverage with highly acceptable properties. The solid beverage contained fructooligosaccharides, erythritol, stachyose, resistant dextrin, strawberry juice powder, and vitamin C. The products of the solid beverages with and without *L. reuteri* have the same taste and are packed at 1.5 g per package. *Limosilactobacillus reuteri* has been reported to colonize the intestines of almost all vertebrates and mammals, has good biocompatibility, and is widely used for food production. We previously used targeted sIgA immunomagnetic bead enrichment and separation technology (patent number ZL201610975479. X) to isolate a strain of sIgA-coated *L. reuteri* FN041 (patent strain storage number: GDMCC60546) from Gannan breast milk. This species is included in the list of bacterium strains that can be used for food released by the National Health Commission and is a probiotic that can be used in ordinary food. Genome sequencing analysis revealed that *L. reuteri* FN041 has no horizontally transmissible antibiotic resistance or pathogenic genes.

The selected doses (1 × 10^9^, 5 × 10^9^, and 1 × 10^10^ CFU/d) of *L. reuteri* FN041 in the first stage are in the reported safe range and the optimal dosage will be determined and subsequently applied in the second stage. The World Allergy Organization recommends probiotic administration to pregnant females at high risk of bearing a child with allergy during pregnancy and, in case of breastfeeding, lactation (Fiocchi et al., 2015). Therefore, oral intervention with the respective solid drinks for the recruited subjects in the two stages will start from the gestational age of 32–36 weeks until delivery. The mothers will continue to consume the corresponding solid drinks daily for 6 months, from 1 to 3 days after delivery, during the lactation period. All the solid drinks are suggested to be taken orally at bedtime once daily.

#### Criteria for discontinuing or modifying allocated interventions

Participants will be allowed to withdraw from the research at any time without consequences.

#### Strategies to improve adherence to interventions

The project team comprises pediatricians, dermatologists, pediatric nurses, and clinical researchers. This study will ensure that the health or interests of the research subjects are not harmed. We will acknowledge the support of research participants to gain active cooperation. At the beginning of the project, technical training will be provided to the researchers, and specific researchers will regularly contact the subjects to record data. Researchers will regularly follow up, promptly inquire, and collect information on changes in the physical condition of pregnant females and infants and ensure the consumption of probiotics. A mobile app will be developed to strengthen the interaction between researchers and subjects. Participants who fail to complete the entire experimental process will be inquired about their reasons. To ensure the reliability of the results, every effort should be made to keep the number of dropouts below 10%; otherwise, additional research will have to be conducted.

#### Relevant concomitant care permitted or prohibited during the trial

During the research period, neither mothers nor infants will use any probiotic products other than those used in this experiment. Mothers will be encouraged to breastfeed their infants and will be provided with the necessary medical guidance.

#### Allocation and blinding

The participants recruited in this study will be grouped randomly in the first stage of project implementation so that they are consistent with each group according to their age, economic situation, educational level, and other influencing factors.

In the first stage, both the on-site and laboratory analyses will be conducted using a double-blind method. The samples of *L. reuteri* and placebo used in the experiment will be processed and produced by a third-party company. The project organizer, researchers, and participants will not be informed about the intervention methods they receive. The biological samples analyzed by laboratory technicians (including blood, breast milk, and feces) will be blinded to their specific sources and grouping situations until the end of all experiments.

In the second stage, a multicenter cohort study will be conducted, in which the recruited subjects will not be blinded because this stage has been designed to explore the efficacy of maternal intervention with *L. reuteri* in preventing infantile AD.

### Statistical methods

The data obtained in this study can be divided into continuous and categorical variables, which are presented as the mean ± standard deviation or median with interquartile range and frequency with percentage, respectively. Continuous variables will be analyzed for differences among the groups using ANOVA, whereas categorical variables will be analyzed using the chi-squared test. A two-sided *p-*value of <0.05 will be considered statistically significant.

### Participant timeline

**Figure 1:**
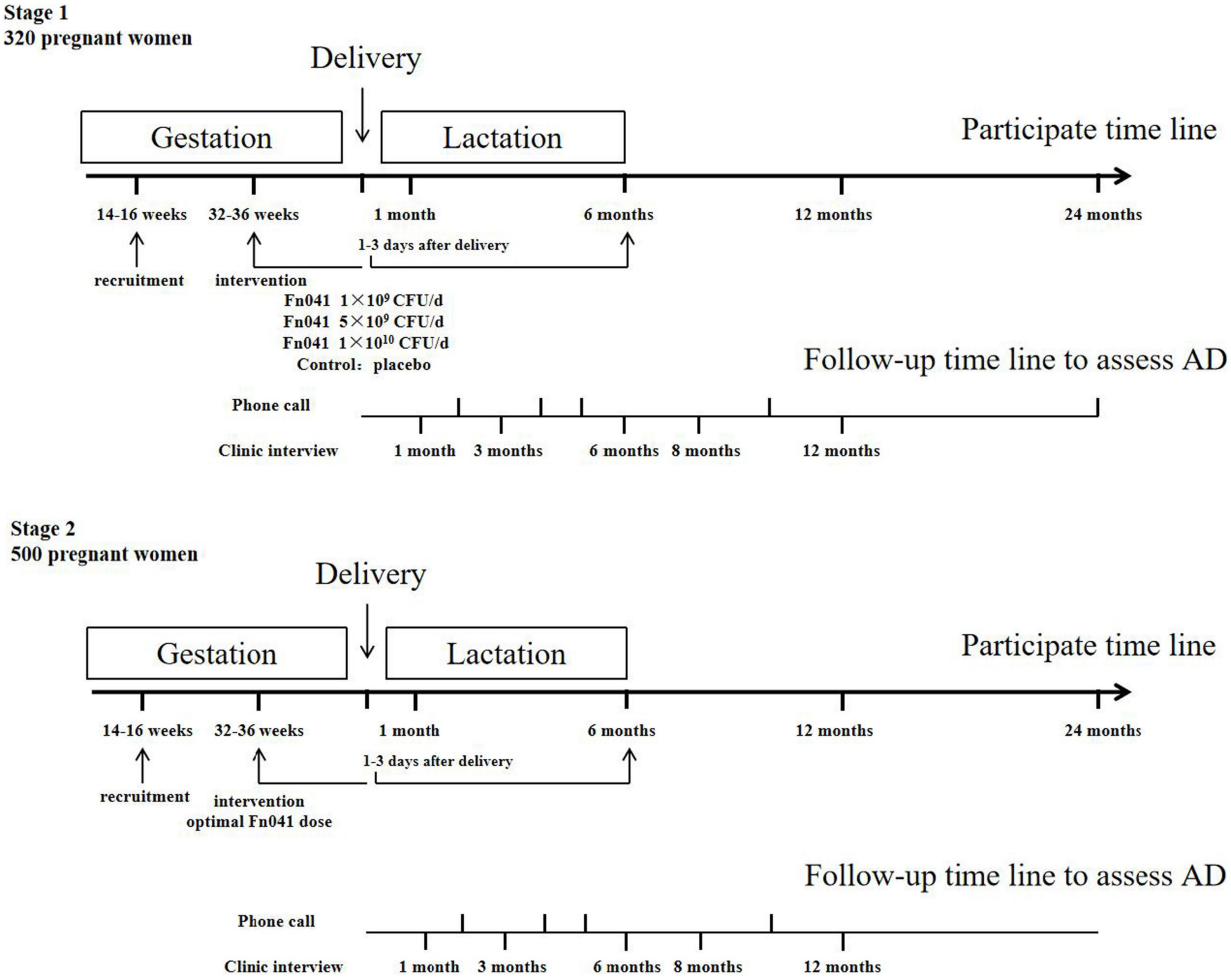
Timeline of the study.

Pregnant females will be recruited at 14–16 weeks of gestation and receive intervention strategy at 32–36 weeks of gestation. Their infants will be followed-up for 2 years after birth. Clinical follow-up will be conducted in the hospital at 1, 3, 6, 8, and 12 months of age, and telephone follow-up will be conducted at 2, 4, 5, 10, and 24 months of age. Two specific pediatric attending doctors are responsible for the clinical follow-up of the infants. Infants suspected of having AD will be referred to the pediatric dermatology clinic, and two designated pediatric dermatologists will jointly diagnose and assess disease severity. Questionnaire surveys will be completed at each clinical and telephone follow-up. Parents will be instructed to contact the project team immediately and take the infant to two designated pediatric dermatologists for diagnosis and treatment if the baby is suspected of developing AD during 0–2 years of age. A detailed flowchart of the study is shown in Figure 1.

### Data collection and sample collection

Clinical follow-up of the infants will be conducted at 7 and 42 days of age, and telephone follow-up will be conducted at 6, 12, and 24 months of age. Questionnaire surveys will be completed at each clinical and telephone follow-up. The infant-specific questionnaire is designed based on the International Study on Asthma and Allergy in Children. Detailed information on birth parity, number of births (single or multiple births), gestational age, mode of delivery, weight (including birth weight), length (including birth length), feeding mode (breast, formula-, or mixed-fed), maternal and infant exposure to antibiotics and probiotics, parental smoking, family history of allergic diseases, infection, and other potential confounding factors will be recorded.

The mother’s feces will be collected within 1 week before intervention with solid beverage and the first week after delivery, whereas breast milk samples (approximately 5–10 mL each time) will be collected at 1–6 (colostrum), 7–15 (transitional milk), and 16–30 (mature milk) d after childbirth. Cord blood will be collected at the time of birth. Fecal samples (approximately 0.5–6 g each time) will be collected from infants in the colostrum, during transitional lactation, and at the ages of 1, 6, and 12 months after birth.

The maternal dietary survey is designed to be carried out before and after probiotic intervention (that is, 6 months after delivery). The dietary and nutritional status survey will be conducted using a 24-h dietary recall method to investigate the intake of various foods by the study participants within 3 days. Their energy and nutrient intake will be analyzed. The food frequency questionnaire will be used to collect information on the types and frequencies of food intake among all study participants and evaluate their dietary structure.

### Outcomes

#### Primary outcomes

In both stages of the trial, the cumulative incidence of AD within 2 years of delivery will be analyzed as the primary outcome. The diagnosis of AD is based on the Williams standard, as previously described, which is widely applicable to outpatient and general population epidemiological investigations in research (Williams et al., 1994). Skin itching is the primary criterion. The secondary criteria are as follows: (1) history of skin involvement on the flexor side, including the cubital fossa, cochineal fossa, anterior malleolus, and neck; (2) history of asthma, allergic rhinitis, or AD in first-degree relatives of children under 4 years of age; (3) history of dry skin throughout the body; (4) eczema on the flexed side (on the cheek or forehead and limbs on the extended side in children under 4 years of age); and (5) onset before the age of 2 years. Infants who meet the main criteria, along with three or more secondary criteria, will be diagnosed with AD. Once diagnosed with AD, disease severity will be assessed using the SCORAD index. Accordingly, infants will be divided into mild (SCORAD: 0–24 points), moderate (SCORAD: 25–50 points), and severe (SCORAD: >50 points) groups (Oranje et al., 2007).

#### Secondary outcomes

The severity of gastrointestinal and respiratory symptoms will be analyzed as a secondary outcome. Meanwhile, regulatory T cells in umbilical cord blood at birth (*n* = 20 for each group) will be analyzed via flow cytometry and the levels of transforming growth factor-β, interleukin (IL)-10, IL-12, IL-13, IL-4, interferon-γ (IFN-γ), tumor necrosis factor-α, and total IgE will be determined using enzyme-linked immunosorbent assay. Further, colostrum and 42-day mature milk level of transforming growth factor-β and IL-10 will be tested. Moreover, the relative abundance of sIgA-coated *L. reuteri* FN041 will be analyzed in breast milk and infant feces using strain-specific PCR, depending on the genome sequence information. *Lactobacillus* strains will be isolated and determined using 16 s rRNA sequencing, metagenomic sequencing will be performed to analyze *L. reuteri* FN041 (*n* = 10 for each group), and StrainPhlAn3 will be used to analyze the data at the strain level (Walsh et al., 2017).

## Discussion

Given that AD is a common pediatric skin disease with an increasing economic burden and limitations of clinical medication, there is an urgent need to develop new strategies for disease prevention in infants. Studies on the gut–skin axis have revealed the key role of gut microbiome dysbiosis in the development of immune-mediated AD (Mahmud et al., 2022). Supplementing mothers with probiotics during late pregnancy and lactation is effective in preventing AD in their offspring, whereas supplementing infants alone with probiotics may not be effective in alleviating AD (Baldassarre et al., 2018). Probiotic supplementation during pregnancy increases the IFN-γ level in cord blood and immunomodulatory factors in breast milk (Prescott et al., 2008). Infants may develop AD when they have impaired IFN-γ activation, which disrupts the Th1/Th2 balance and leads to AD (Herberth et al., 2010; Brar and Leung, 2016). Studies have also revealed that human breast milk is a source of commensal bacteria that are beneficial for the gut health of offspring (Lyons et al., 2020). Gut-specific strict anaerobes and facultative anaerobic bacteria such as *Bifidobacteria* and *Lactobacilli* can be detected in breast milk (Gronlund et al., 2007). Two IgA molecules in the intestinal tract bind to each other through secreted fragments to form sIgA, which can wrap around bacteria in the intestinal tract (Rochereau et al., 2013). sIgA-coated bacteria can be transported into intestinal Peyer’s patches and carried by dendritic cells to the mammary glands (Macpherson and Uhr, 2004).

The mechanism of probiotic transmission from mothers to infants remains unclear, which hinders the isolation, screening, and application of probiotic strains for AD prevention. We previously observed that sIgA-coated bacteria are key functional bacteria for vertical transmission between mother and child and that breast milk is a good source of sIgA-coated probiotics (Cui et al., 2020; Ding M. et al., 2022). *Limosilactobacillus reuteri* is a well-studied probiotic bacterium detected in breast milk (Mu et al., 2018). Previous studies have reported the therapeutic potential of *L. reuteri* in the management of allergic diseases (Forsythe et al., 2007; Karimi et al., 2009). Using a patented technology developed by our group, sIgA-coated *L. reuteri* FN041 was isolated. Preclinical experiments have confirmed the therapeutic potential of *L. reuteri* FN041 in preventing AD (Qi et al., 2021; Zhou et al., 2022). Maternal mice supplemented with *L. reuteri* FN041 can promote the expression of intestinal antimicrobial peptides and enhance the mucosal barrier function and intestinal sIgA production by remodeling the gut microbiota in their offspring (Qi et al., 2021).

A previous study showed that no adverse reactions were observed in individuals supplemented with live *L. reuteri* at a daily dose of 1 × 10^8^ to 1 × 10^10^ CFU (Mobini et al., 2017). A meta-analysis of eight randomized controlled trials of probiotic application in >1,500 pregnant females also revealed that probiotic consumption from 32 to 36 weeks of gestation did not increase the incidence of abortion or malformations and had no effect on birth weight, gestational age, or delivery mode (Dugoua et al., 2009). In the first stage of this clinical trial, we will assess the safety of maternal *L. reuteri* FN041 supplementation. We aim to determine the optimal dose of maternal *L. reuteri* FN041 during pregnancy and lactation for infantile AD prevention. In the second stage of the study, we intend to promote the application of maternal *L. reuteri* FN041 supplementation in a multicenter setting to evaluate the incidence of AD after the intervention. In addition, *L. reuteri* FN041 in breast milk and infant feces, detected using metagenomic sequencing and StrainPhlAn3 analysis, indicate that a specific strain *L. reuteri* FN041 can be transmitted to infants through breast milk, providing new evidence for vertical transmission in maternal and infant microbiomes.

Importantly, previous clinical studies have confirmed the protective action of a mixture of probiotics against AD. Here, we aim to identify the potential role of a more efficient single strain of *L. reuteri* FN041 in AD prevention. We have discovered that its ability to adhere to mucus is far superior to that of LGG and *L. reuteri* DSM17938 (unpublished data), which may explain its better preventive effect against AD than LGG and *L. reuteri* DSM17938 in an established AD mouse model (Zhou et al., 2022).

In addition, the regulations regarding the consumption of probiotics by infants in China are strict. Currently, only 14 strains of probiotics are allowed for infants under 1 year of age, and the approval of these 14 strains for infant consumption is mainly based on safety considerations rather than functional perspectives. Notably, all commercial strains used to prevent AD in clinical practice are isolated from the intestines of Western adults, and China has not yet developed probiotics for preventing AD. Therefore, there is an urgent need to identify probiotics in domestic breast milk that can effectively prevent infantile AD. The results of this clinical trial provide evidence for the efficacy of *L. reuteri* strain FN041 isolated from breast milk in preventing or curing AD in infants and provide practical advice for the supplementation of specific probiotics for the primary prevention of AD in pregnant females. Because the immunological mechanisms of AD are similar to those of other allergic diseases, understanding the mechanisms of breast milk-derived probiotic strains can contribute to their application in maternal nutritional intervention strategies to prevent diseases caused by the interaction between the infant intestinal microbiota and the immune system.

### Trial status

This study is in its first stage, from October 2022 to December 2025. Subject recruitment in the first stage commenced from July 2023 and will be continued till December 2023. Subject recruitment in the second stage will start from July 2024 to December 2024.

## Data availability statement

The original contributions presented in the study are included in the article/supplementary material, further inquiries can be directed to the corresponding authors.

## Author contributions

RY: Conceptualization, Funding acquisition, Writing – original draft. YM: Funding acquisition, Writing – original draft. ZL: Conceptualization, Writing – original draft. CQ: Methodology, Supervision, Validation, Writing – review & editing. AX: Methodology, Writing – review & editing. YJ: Software, Writing – review & editing. BZ: Project administration, Resources, Supervision, Visualization, Writing – review & editing. JS: Conceptualization, Methodology, Supervision, Writing – review & editing.

## Footnotes

^1^ http://powerandsamplesize.com/Calculators/Compare-2-Proportions/2-Sample-1-Sided

## Abbreviations

AD: Atopic dermatitis
*L. reuteri*: *Limosilactobacillus reuteri*
RCT: randomized controlled trial
HN001: *Lactobacillus rhamnosus* HN001
LGG: *Lactobacillus rhamnosus* GG
sIgA: secretory immunoglobulin A
IFN-γ: interferon-γ
Tregs: regulatory T cells
IL: interleukin
